# Deep Cross-Cultural Reconstruction Process in the Context of Cultural Potential: A Qualitative Study

**DOI:** 10.3389/fpsyg.2022.727616

**Published:** 2022-05-06

**Authors:** Fengyi Zhang, Jinyu Xie, Ling Luo

**Affiliations:** Business School, Sichuan University, Chengdu, China

**Keywords:** deep cross-cultural reconstruction process, cross-cultural adaptation, reconstruction strategy, cultural integration model, ladder model

## Abstract

Cross-cultural adaptation significantly impacts the development of individuals in different cultural environments. Because of art scholars’ intense cultural intervention, their cross-cultural process—cultural reconstruction—significantly differs from that of other groups. However, there is insufficient research on their process. In this study, 29 contemporary Chinese artists living in Germany were interviewed. Grounded theory was used to propose a new ladder theory. We found that the cross-cultural reconstruction process includes two sub-processes and seven ladder-like stages containing the “driver–strategy–outcome” logic. Arguably, the ladder model provides a stronger explanation for the mechanism of art scholars’ cross-cultural reconstruction process.

## Introduction

Studying, working, and emigrating abroad have become more common at present. However, when destination and home countries’ cultures differ significantly, the problem of cross-cultural adaptation emerges. If adaptation does not go well, it may restrict individuals’ development. Cross-cultural adaptation is the process of learning and adapting to a new culture (Berry, 2003). While adapting to heterogeneous cultures, cross-cultural individuals undergo a series of psychological and behavioral adjustments and development processes, seeking to balance continuous exploration and consolidation of their findings (Ward and Szabo, 2019). The psychological and emotional changes experienced by such individuals in adapting to host cultures described and explained in the famous U-shaped (Oberg, 1960) and W-shaped models (Gullahorn and Gullahorn, 1963) provide the basis for this study.

Cross-cultural adaptation studies across a range of disciplines have focused on immigrants, government-sponsored personnel, and students. Accordingly, the degree of cultural intervention—the depth of cultural participation and involvement—has been studied at a social-cultural level, including scope of adaptation to the physical environment, conditions of social-material life, economic conditions, social systems, interpersonal communication, and cultural norms and practices related to life and work (Kim, 2017). However, because of their special cultural interests, art scholars, an understudied group, experience adaptation processes during deep integration, remodeling, and reconstruction of two completely different cultures—assimilating into a social culture and internalizing intellectual culture (philosophy, literature, and art) and learning and deepening culture. Deep cultural construction in artists will involve deep thinking triggered by the learning process of exposure and internalization of philosophy, literature and art, as well as changes in cultural cognition, values, thinking mode, creation mode and content, which will in turn affect the audience of their artistic works. This study reveals that the art scholars’ mental and intellectual adaptation tasks are intensive, and their strong willingness to reconstruct culture separates them fundamentally from other cross-cultural people.

The creative nature of artists’ work requires them to go deep into the mental source of their creation. The artists we studied believe that the way to really understand your cultural background is not to immerse yourself in your home culture, but to go “outside,” interact with other cultures, and receive the “rebound” of the home culture’s collision with other cultures, assimilating feedback from other cultures in order to carry out cultural cognition. These artists have cross-cultural and direct cultural contact experience, and their original cultural composition has been deconstructed. What they see is not an image of the West processed and created in accordance with their background culture and imagination, as might be seen from China. In the process of constantly searching for creative methods and content, they continue to conduct in-depth exploration of other cultures. Therefore, the single cultural framework is broken through, the cultural vision expanded, the cultural dimension increased, and new cognition and ability generated under the interaction of two or more cultures, also fostering their integration. This cultural power continues to burst forth in the accumulation, review, and reflection of such artists’ cross-cultural experience, which enables them to constantly find inspiration for creation. This paper conceives these events and processes under the umbrella of cultural “reconstruction.”

Cultural differences are unique narrative ways generated and understood by different regions, countries, and nations and their peoples as part of their survival practices. The presuppositions of their intellectual histories are different (Baumeister, 2005). In classic and traditional concepts of culture, every culture has a decided delimitation toward the outside, which ensures it is distinguished and remains separated from other cultures (Welsch, 1999). Complete homogenization of systems of meaning and practice has never occurred in the world before, and is unlikely to happen soon (Hannerz, 1990). Different cultures guide individuals’ thoughts, feelings, and actions (Markus and Conner, 2014), and constantly supply different meanings and practices, which may give rise to systematic cultural variation (Markus and Kitayama, 1991). Also, different features of the sociocultural contexts that people inhabit shape their psychological processes, which in turn reflect and reproduce those sociocultural contexts (Markus and Conner, 2014). As a result, “[t]he cultural inheritance of societies is quite persistent” (Inglehart and Welzel, 2005).

Two major sets of social and cultural tasks comprise by independence and interdependence (Kitayama et al., 2006). From an East–West viewpoint, in the Western cultural framework, independence is much more dominant, which encourages the individuals to see themselves and others as separate entities in the world, as well as a focus on individuality and a human action. In contrast, interdependence is highlighted and salient in the Eastern cultural framework as it encourages individuals to see themselves, others, and objects in relation to their context. Therefore, the artwork is also very different. Eastern art is more about objects, animals and scenery and focuses on the beauty of nature or social harmony, while Western art especially emphasizes people and strives to create a personalized expression. Unlike the open and diverse cultural systems of Europe and the United States, traditional Han culture, rooted in Confucianism and supplemented by Taoism and Buddhism, forms a concentric and radiant culture that deeply influences surrounding cultures. In ancient times, Chinese culture had a high cultural potential and strong centripetal cohesion. In modern times, as Chinese society has been relatively closed and isolated from the wider world, it has fallen into a low-potential cultural circle (Levenson, 2018). The development of contemporary art in China is also relatively backward.

However, with increasingly frequent intercultural communication in the world, “the old homogenizing and separatist idea of cultures has furthermore been surpassed through cultures’ external networking”; cultures’ actual forms, their types of relations, and even the structure of individuals’ identities and lifestyles have been redefined (Welsch, 1999). World cultures have diverse dimensions. As globalization has created unprecedented conditions for new dialogues between cultures, cultural hybridity and creolization—forms of mixing of two or more cultural elements from different and the transformation of cultural relations thereby—have emerged. Moreover, cultural hybridity is also manifested in the lifestyle of immigrants, who distinguish or mix a “home culture” used in the home and a “host culture” used outside (e.g., at school or in the work place; Pieterse, 2009). People also have more opportunities to experience different cultures, and people with different goals, tasks, and jobs will experience different aspects and depths of cultural involvement. Moreover, positive and equal dialogue between China and the West is possible when Chinese are no longer trapped in the preconceptions of Western centrality, but instead see the West from their own perspective (Beck, 2016), which is also the implication of our research.

Since China’s reform and opening up beginning in 1978, the significance of Western art to Chinese art has increased. Global cultural inputs urge contemporary Chinese artists, art students, and art and cultural scholars to consider the development of art beyond the framework of a single culture. This study considers these substantially overlapping categories together, as the participants were generally artists and art scholars as learners in Germany (whether students in China or not). Amid East–West collision in literature, art, philosophy, and aesthetics, they are faced with a complex task of adaptation and integration. Therefore, this group provides an excellent sample for research on the understudied topic of cultural reconstruction.

This study examines cross-cultural adaptation and reconstruction in 29 Chinese contemporary artists who have studied in Germany. Using qualitative research methods, it comprehensively explores the mechanism of cross-cultural reconstruction and builds a new conceptual model which connects specific cross-cultural and cross-national contexts, enriches existing research, and provides a theoretical basis for future studies. The research mainly focuses on two aspects of cross-cultural adaptation: process model and reconstruction strategy.

Cross-cultural adaptation has produced fruitful research in anthropology, sociology, psychology, and communication science. Previous studies have formed three widely recognized models: the U-curve theory, W-curve hypothesis, and stress–adaptation–growth dynamic. The U-curve theory and W-curve theory are based on the study of expatriates and international students. Oberg (1954) advanced U-curve theory based on the culture shock, psychological changes in foreign cultural environment, by dividing the process of cross-cultural adaptation into four stages: honeymoon, crisis, recovery, and complete adjustment. The U-shaped theory describes the process from experiencing anxiety, frustration, and culture shock to the ultimately satisfying adjustment for those who emigrate, due to the loss of all familiar markers and symbols of social interaction. Lysgaard (1955) conducted empirical research on U-curve theory and revised the four stages as honeymoon, culture shock, adjustment, and mastery, highlighting psychological and emotional changes during the process. Gullahorn and Gullahorn (1963) proposed the W-curve hypothesis, extending the cross-cultural adaptation model beyond these four stages to also reflect the time pattern of adapters’ responses and re-adaptation to their own culture upon return. It posits that people who have achieved good adaptation abroad will still face maladjustment after returning home and that they need to re-adapt to their home culture. This is because when a person moves from one social system to another with different value orientations and normative expectation characteristics, that person will tend to acquire expectation patterns that are compatible with their new social system.

Distinct from these linear process studies, the stress–adaptation–growth dynamic analyzes changes in individual behavior in a heterogeneous culture, proposing that individuals adapt to a heterogeneous cultural environment to establish a work-and-life-based relationship with it. This process stems from people’s natural desire to achieve an internal balance in an unfavorable environment through two interrelated sub-processes: acculturation and deculturation (Kim, 2008). Pressured between demands for and resistance against cultural adaptation, individuals undergo a spiral development by constantly “drawing-back-to-leap.” Beginning with mental and physiological dislocation and stressful experiences, through continuous learning about new cultures, people improve their functional level and psychological efficiency over time and finally fully adapt (Kim, 2017).

The U-curve and W-curve process models suggest that the adapter is always an outsider whose adaptation process is psychological adjustment and establishing two sets of independent behavior models. The U-curve entails a change of attitude toward external environments, while the W-curve involves resocialization. In these two models, the objects of acculturation are mainly the social system and cultural conventions of the host country, and individuals’ sense of identity depends on whether they are satisfied with their communication with the new society. In the process of acculturation, the main content changed is one’s own social role. In terms of objects, these two models lack an element of involvement in pure culture (e.g., philosophy, literature, and art). In terms of identity standards, there is a lack of discussion on in-depth intervention in and reflection on Chinese and Western culture; in terms of changing content, there is a lack of discussion on the transformation of individuals’ own cultural formation and their output and expression of culture.

Assimilation is essential for the stress–adaptation–growth dynamic, which requires adapters to remove themselves from the home culture and form the ability to communicate and behave following host-country norms (Kim, 2017).

Other recent studies on cross-cultural adaptation include nuanced views of Chinese international students’ acculturation and adaptation (Quan et al., 2016; Heng, 2018) have broadened classical theory to encompass the distinctive nature of Chinese students and factors that influence their adaptation to studying abroad (Crawford and Wang, 2015; Xie et al., 2020). However, research has not taken art scholars as its object, nor has it tackled the issue of cross-cultural reconstruction.

In cross-cultural adaptation, individuals encountering cross-cultural pressure or conflict adjust attitudes and behaviors, including willingness to adapt. Scholars have summarized several adaptation strategies depending on different orientations: Berry (1976) proposed adjustment, reaction, and withdrawal as general adaptation strategies; Lazarus and Folkman (1984) proposed problem-centered and emotion-centered coping styles; and Endler and Parker (1990) added a third coping style: avoidance. Acculturation overlaps with adaptation. From the perspective of values and maintaining one’s own ethnic identity (heritage-cultural orientation) or desiring contact with different cultures (dominant-cultural orientation), Berry et al. (1989) formulated an acculturation strategies model comprising both strategies and results, with four choices, assimilation, integration, separation, and marginalization, based on the conceptualization of acculturation as a two-directional, multidomain, and complex process. Worth mentioning is that biculturalism is closely intertwined with acculturation, being one of four acculturation ways and involving individuals using the integration strategy, maintaining an orientation to both cultures (Nguyen and Benet-Martínez, 2013). However, Bicultural individuals do not constitute a homogeneous group (Nguyen and Benet-Martínez, 2013). According to the view of cultural norm orientation, bicultural individuals are those who internalize two cultural systems and are guided by two cultural norms in different cultural situations, and this internalization profoundly affects their thinking, emotions, and behavior (Hong et al., 2000). They identify with and become involved in both the host culture and their home culture (Berry, 2003). This type has a dynamic of transformation between two cultural frameworks that remain separate, rather than a dynamic of cultural formation. The experience-oriented view holds that bicultural individuals are those individuals who have studied or experienced two cultures and languages for a long time and have the experience of living in two cultural contexts (Hong, 2009). This definition does not emphasize cultural identity or cultural internalization, and posits that people with lived experience and cultural learning in two cultures can be called bicultural individuals.

There are also several models that have discussed what happens to individuals as they undergo the process of second-culture involvement, as well as describing the psychological processes and cultural experiences associated with being bicultural. Assimilation models assume that individuals of one culture relinquish their home cultural identity as they acquire a new identity in a different culture (Tadmor et al., 2012), as a result of which they experience a sense of alienation and isolation until they have been accepted within the new culture. Acculturation models assume that individuals are forced to learn the new culture and will always be identified as a member of their home culture, even though they have become a competent participant in their new culture (LaFromboise et al., 1993). Alternation models posit that individuals should be able to understand two different cultures and change their behavior to fit a particular social context, and as a result they could have a sense of belonging to two different cultures without having to choose between them and compromising their sense of cultural identity (LaFromboise et al., 1993). Multicultural models argue that individuals can maintain a positive identity as a member of their home culture, and at the same time, can also develop a positive identity by engaging with a larger entity consisting of several different cultural groups. Their public and private identities are disconnected, and bicultural stress could lead to personal growth (LaFromboise et al., 1993). Fusion models posit that cultures will fuse together to form a new culture. Individuals would have experiences similar to those undergoing assimilation. Once fused, the psychological reality would be indistinguishable from that of members of the second-culture under mutual influence (LaFromboise et al., 1993). In either case, bicultural individuals may enhance greater integrative complexity, intellectual flexibility, and creativity in the process of negotiating two cultures (Benet-Martínez et al., 2006). This study will discuss another phenomenon and group within biculturalism, the group that deeply involves itself with and internalizes intellectual culture and is involved in cultural reconstruction and cultural formation.

Profound cultural intervention and reconstruction helps individuals to profit from globalization. Art scholars naturally tend to be deeply involved in cultural matters of home and host countries. This study fills these gaps in research participants, in-depth discussion of cultural reconstruction, and consideration of the inequality of cultural situations by proposing the innovative ladder theory of cross-cultural reconstruction. Exploring this issue from the viewpoint of Chinese art scholars will deepen cross-cultural theoretical research and help guide cross-cultural practice of individual adaptation and cultural reconstruction. First, this research studies the cultural reconstruction process experienced by people who have moved across the East–West cultural divide under different scenarios of potential cultural energy. Second, it examines whether there are special motivations and/or adaptation and reconstruction strategies in different cultural reconstruction stages. Third, it builds a process model for art scholars’ deep cross-cultural reconstruction.

Cross-cultural studies have traditionally only studied cultural adaptation instead of the difficult topic of deep-seated cross-cultural reconstruction. Deep-seated cross-cultural reconstruction is an essential change of individual cultural formation in a cross-cultural context. It takes people themselves rather than the general culture as the main body, focusing on changes in individuals depending on cultural context. The reconstruction behavior centers on the interaction between people and culture, focuses on the development of individuals in culture, and reconstructs their own cultural formation—reconciling the inner world of consciousness with the outer world of human interaction-in-culture. It is a process by which individuals break through the single cultural framework, expand their own cultural volume, and reconstruct their own culture formation by learning and skillfully using culture for cultural creations (Epstein, 2009). That is, it is the endogenous transformation of their cultural consciousness. Cross-cultural reconstruction is not only a kind of cultural consciousness but also a kind of behavior proposition. Its characteristics are as follows: first, it respects the particularity of each culture; second, it does not deny the individual’s own indigenous substrate inherited from history; third, it expands cultural identity from home culture to other heterogeneous cultures; fourth, each individual can decide and reconstruct their own cultural formation; fifth, biculturalism is not the end of cross-cultural reconstruction, but takes different cultures as a medium to open up the possibility of integrating even more cultures.

Moreover, art scholars engaged in intellectual cultural learning and reconstruction have not been included. Consequently, existing research offers limited understanding of cultural reconstruction’s root causes. The degree of cultural intervention previously remained at the level of social culture, such that changes in geography and cultural context could generate significant differences in individual psychology and emotions. The reasons for maladaptation include poor conditions of social-material life brought about by their limited economic ability, poor communication, different customs, and lack of alignment between an individual and their expectations of roles in a different culture, involving life- and work-related areas such as physical environment, material conditions, social system, interpersonal communication, and cultural norms and practices. To grow into a social person in one’s home culture is the first instance of socialization, and to adapt to the social conventions and cultural customs of another culture and play an expected role is then resocialization. Resocialization in a strange environment changes adapters’ emotions and explicit behaviors, re-positions their cognition, internalizes expectations suitable for their new social system, and finally achieves adaptation. Art scholars must face the difficulties of general adapters and also resolve the reconstruction problems caused by the vast differences between their home culture and the strong, heterogeneous Western culture in their cultural composition and professional practice and habitus. This issue has not been addressed in previous research.

Previous process models of cross-cultural adaptation were based on life and work and did not explore the adaptation and reconstruction process in the mental and intellectual fields of those who experience deeper cultural learning. Additionally, studies on U-curve and W-curve theory did not clearly explain the mechanism, the driving force, strategies, or cause and effect used by reconstructors at each stage to resolve deep cross-cultural puzzles; they instead focused more on using a hypothesis to describe the phenomenon. Although the stress–adaptation–growth dynamic explains the adaptation process, it does not explain in detail the different levels or types of stress faced. Furthermore, it generalizes the mechanism by not describing the stages. Moreover, in cross-cultural adaptation studies, whether for general pressure in daily life or value-related significant pressure, the strategies for coping with pressure are mostly comprehensive ones adopted for the whole process of adaptation, rather than specific to each stage.

Earlier research has been mainly based on the Western context, where movement is often between two strong cultures, or from a strong culture to a country with relatively lower cultural influence (Oberg, 1960; Gullahorn and Gullahorn, 1963; Kim, 2017), as represented by studies on U-curve theory, W-curve hypothesis, and the stress–adaptation–growth dynamic between them. These models are unsuitable for two countries that are culturally very different from one another. With the academic community’s increasing attention to overseas student groups, there have been many recent adaptation studies of students from developing countries studying in developed countries. However, this literature is mostly limited to the life- and work-related adaptations of overseas non-art learners at a social-cultural level (Smith and Khawaja, 2011), without involving adaptation and remodeling in the context of the intellectual and cultural realm and thus failing to reflect the particularity when individuals cross a long cultural distance.

This study of cross-cultural reconstruction is a new reflection on previous work on cross-cultural adaptation; however, the current focus on cultural and art scholars’ cross-cultural reconstruction of cultural formation makes an important new contribution to the field of cross-cultural adaptation. With the deepening of globalization, the height and depth of cultural dissemination and exchange have shown unprecedented vitality between China and the world; hence, cross-cultural issues are no longer confined to ordinary knowledge learning or to working and living in another country for Chinese art scholars. Deeper cultural intervention and reconstruction are becoming increasingly prominent, stimulated by the opportunities of the era and driven by individuals seeking development. This is an issue that needs to be explored. The problem of cultural reconstruction faced by Chinese art scholars in different scenarios of potential cultural energy is exactly what more cross-cultural individuals will confront in the near future. They not only constantly update their own cultural formation in the cross-cultural context but also carry out creative expression and communication of cultural resources. This phenomenon expands beyond the scope of previous discussions on cross-cultural adaptation and biculturalism into deep intellectual cultural involvement and art scholars’ reconstruction.

## Materials and Methods

This study will use grounded theory for exploratory theoretical construction. Qualitative research is suitable for presenting a detailed process and explaining its formation mechanism; grounded theory is suitable for reflecting on social phenomena associated with a specific situation and then analyzing the data collected, considering important problems of the target group, and on that basis constructing a theoretical account of a phenomenon (Glaser and Strauss, 1967)—generating a new theory from data analysis, induction, coding, construction, and verification (Strauss and Corbin, 1998; Charmaz, 2014).

Generally, the grounded theory researcher first determines the research problem, then encodes, analyzes, and models data connected to the phenomenon, and finally derives relevant concepts and theories.

An ethics approval was not required as per institutional guidelines and national laws and regulations. We just conducted in-depth interviews and were exempt from further ethics board approval since this research did not involve human clinical trials or animal experiments. All participants gave written informed consent in accordance with the Declaration of Helsinki. Research respondents were ensured confidentiality and anonymity. All participation was voluntary.

### Participants

Our interest in this subject was driven by the fact that going abroad is becoming increasingly common. On the basis of reading the literature, we consider whether there are other deeper cultural exchanges and deeper changes brought by culture to individuals in addition to the established aspects and modes of cross-cultural adaptation. Therefore, we selected jobs where culture is the whole job content. Based on the homogeneity requirements of qualitative research samples, we focused on artists and art scholars because they go abroad to study culture deeply, conduct art research, and create culture at the same time, and focused on their cross-cultural adaptation.

After returning to China, a number of them are engaged in art education. Unlike other types of scholars who study objective things, the job of an artist has a particular strength, breadth, and depth in terms of cultural intervention. First, culture is what they do, and self-reconstruction is how they do it. Artists’ understanding of culture is highly personal given the nature of their practice: they express and externalize the creative characteristics of cultural resources within their own artistic and creative thinking, and provide “meta-ideas” for the cultural industry (Romer, 1993). In addition, as mental products, artistic works have a distinct individual uniqueness and character and can only be completed by artists themselves. Influenced by factors such as individual artistic concept, cultural ability, original cultural background, and surrounding cultural environment, the growth rate and artistic level of artists will differ greatly. Second, in cross-cultural context, these Chinese artists showed a desire to promote cultural exchange and to interpret the essence of Chinese culture for outsiders in a way that the world can understand. In the process of artistic creation, it is also necessary to have deep thinking and deep understanding of different cultures. They need to be open-minded and inclusive, draw inspiration from different cultures, and incorporate different ways of thinking. They try to create bridges connecting Chinese and other cultures.

Obtained through purposive sampling, the sample comprised 29 contemporary artists featured in the exhibition “Complement and Accord: Works Exhibition of Chinese Artists Studying in Germany.” Based on previous studies, given our use of a purposive sample, 29 participants could achieve theoretical saturation. They had come to study in Germany after China’s reform and opening up, beginning in 1978. During their university years, they had received a complete undergraduate art education in China. After graduation, they had become professional artists, a group covering <10% of their respective graduating classes. They had formed a relatively mature personal art style—the expression of an external imprint through a work of art that can be relatively stable, more profound, and more essential, to reflect the artist’s personal mental temperament, aesthetic concept, aesthetic interest, aesthetic ideal, and other internal characteristics. Simultaneously, these art scholars glimpsed Western avant-garde art ideas and works, giving them an initial yearning for Western art. Later, they chose a different path from that of other artists, who stayed in China: cross-cultural learning. Owing to the overseas study mechanism, they passed through the screening of the domestic art system and market and became highly successful artists.

Participants were selected for this study based on the fact that an accurate representation of the complexity of the process requires work with those who have undergone intellectual cultural adaptation and reconstruction and have been intensely involved in the culture over an extended period of time. This sample represents a group of individuals who underwent intercultural training from 1982 to 2009—a time span of nearly 30 years. This is a relatively long historical period for study abroad, thoroughly covering the various stages of intercultural experiences, phenomena, and problems during this process. Therefore, the sample accurately reflects the phases of cultural reconstruction and is suitable to represent the entire process of cross-cultural reconstruction. Moreover, the time span is sufficient to observe the impact of cross-cultural reconstruction. These artists are highly rated in the contemporary Chinese art system and enjoy a great following, as evidenced by such factors as solo shows, invitations to present their work, and being warmly welcomed by audiences, curators and art institutions. Therefore, the cross-cultural reconstruction distinguishes them from other groups because of their influence and creativity in literature and art as well as their understanding and sublimation in thought and spirit, which is particularly important for this group as a practical guide, according to the data.

Germany opened up to China relatively early. Germany’s education system was opened to Chinese learners even before China’s reform and opening up, and the well-established channels brought many Chinese students into Germany (Ji, 2004); they then greatly impacted various fields after returning home, immensely promoting the construction and development of modern China (Ye, 2005). Germany is an important hub for post-war contemporary art in Europe, surpassing France in influence (Ji, 2004); after the reform and opening up, many Chinese students developed a yearning for Germany due to the influence of German art styles such as expressionism, and early German exhibitions in China, this was mentioned by almost all the interviewees. Contemporary German artists such as Joseph Beuys, Jörg Immendorf, Gerhard Richter, Anselm Kiefer, and Georg Baselitz deeply influence Chinese artists. Whereas most artists who studied in France, the United States, and other countries become independent artists and rarely enter the art education system, the artists who studied in Germany—armed with German reasoning and methods and encouraged by German examples and practices—reflect their thoughts on art and promote art education in China (Ye, 2005). Therefore, by focusing on Germany as a country with a strong influence and specific culture, one can draw reliable conclusions for cross-cultural reconstruction processes and the role of deep cultural intervention.

This sample of artists/art scholars is unique because:

The participants’ study and job contents are different from those of immigrants, government-sponsored personnel, and students who study science, industry, agriculture, commerce, and medicine. Artists learn philosophy and literature, but they do not study theories or conduct further theoretical research as do scholars majoring in philosophy and literature. Instead, they transform their deep thinking on these topics into works of art.

Their purposes are also different: scholars in sociology and anthropology believe that the purpose of cross-cultural adaptation is to facilitate integration into the host country. By contrast, scholars in communication believe that cross-cultural adaptation serves to facilitate communication and interaction. In contrast, the artists in this paper assimilate in order to reconstruct their own culture so as to create more works with profound cultural significance.

In addition, they behave differently, mainly in their strategies: pressured by cultural reconstruction’s demands (the “push” of host cultures) and resistance (the “pull” of the home culture), their strategies involved adapting to host cultures and reconstructing their home culture. These two strategies, including several specific sub-strategies, are presented in the results and have not been covered by previous study populations.

### Data Collection

We collected first-hand data using 35 semi-structured in-depth interviews between November 2016 and September 2017. Some artists were interviewed twice; particularly for those artists who were interviewed in the early stage, a supplementary interview was conducted after modifying the interview outline, and the generated transcripts amounted to nearly 610,000 words (see Supplementary Table 1 for information on the interviewees). The interview was conducted in Chinese in artists’ own studios or exhibition spaces.

The initial interview questionnaire was designed on the basis of a literature review. The interviewees were mainly asked to narrate retrospective stories of their cross-cultural experiences, but were guided to answer several key questions covering their motivations for cross-cultural study, what difficulties they encountered in the process of cross-cultural adaptation, what factors helped or hindered their cross-cultural adaptation, the important others in this adaptation, and what methods helped them better adapt to the host culture. These questions were designed to trigger interviewees’ “episodic memory” to enhance data accuracy (Eisenhardt, 1989). By interacting with the research participants, we could interpret their behavior and sense-making.

We audio-recorded the entire interview process, with interviewees’ written consent. Within 24 h after each interview, we sorted the interview data, wrote the interview notes (Yin, 1984), and transcribed the original recording. When analysis of new data no longer generated additional categories, theoretical saturation was considered to have been achieved, and data collection was stopped when a category supplied data of sufficient depth and width to understand a phenomenon and its relationship with other categories.

### Data Analyses

The data analysis and collection stages were conducted simultaneously. After transcribing the interviews, we read and analyzed the verbatim transcription in detail. Based on grounded theory coding, data analysis was divided into three stages: open coding, axial coding, and selective coding. We adopted various coding strategies in the coding process. Simultaneously, we wrote memos to record the idea-generating process and help us rethink and generate ideas and codes (Glaser, 1978). We also used continuous comparison to improve the fitness of the original text to concepts and categories (Strauss and Corbin, 1998; Charmaz, 2014). Finally, we used NVivo 11 Plus as an auxiliary coding tool.

We used open coding on the initial interviews. Participants mentioned issues that differed from those identified in the existing literature on cross-cultural adaptation. Specifically, participants mentioned the origins of desire to study abroad:

*At that time, we didn’t know Western modernity at all. The teacher probably stopped talking about Pop Art. After that, we didn’t know what it was, neither did the professor, nor did the students. When I was getting ready for graduation, I drew two sets of works: one set is the standard works of the school; the other group was my favorite works—they were black and white. I was deeply shocked that the professors very frankly and sincerely said that they do not understand this, and they cannot give me any comments. Then who can understand that and where? So I desire to study contemporary art in Western countries. (SZ)*

Experiencing deeper culture shock:

*When I got there, I saw the students’ work in the museum and in the university, and I realized that I hadn’t seen that before. It was a completely different concept from what I had studied in college, because at that time we were studying the early Western neoclassical [form], and then, impressionism. (LYG)*

Feeling lost and stranded:

*In the second year, I began to feel miserable. I felt neither drawing big nor small paintings is appropriate and comfortable, so I kept on doing all kinds of paintings aimlessly. (CL)*

There was also a sense of cultural inferiority:

*Just like a tower, the view from the top is different from the view from the bottom. Others see the view from the seventh floor, but we only see the view from the second floor. We think we have reached the top after climbing one floor, but it is not at all, so we feel very inferior at that time. (SZ)*

Feeling unrecognized and “not fitting in”:

*If we want to do something in China, and if we want to be noticed, people are very aware of it, because in this cultural background system, our unique thinking is popular and noticeable, but once we do something in Europe, people don’t really care about us. (DGY)*

However, they valued the cross-cultural experience:

*When we read Kant, we read some ideas, such as the thing in itself, and it was not clear what it meant. When I went to Germany and re-read these questions in German, it was very clear. It may not be so clear to read German till you go to Germany, for it is German soil where it grows. (ZQS)*

For subsequent interviews, we adjusted the content of the questions to be more suitable for artists. This became necessary because our original questions were designed based on the existing cross-cultural research which had not included artists before. Additionally, we continued to add to the sample in an attempt to obtain a complete picture of this phenomenon. We continuously compared the interview materials and literature. Through open coding, we found that there were stages in the cross-cultural adaptation of art scholars. Typical descriptions were “when I first went, then, later, now,” “the first year, second year, third year, 5th–10th years, 20th year,” “just arrived in Germany, the first 3 years, the fifth year, and then later, before returning home,” among others. Therefore, we extend the initial question to our first specific research question, which seeks to understand what the cross-cultural adaptation process of art scholars is and what the adaptation stages are.

Upon analysis of the process stage, we found that it was not enough to describe the problems encountered by art scholars, which also went beyond the scope of the word “adaptation.” Therefore, we thought that “adaptation” was no longer applicable to this group of people, and a more accurate term was needed. Concurrently, we found that in the whole period of their cross-cultural experience, art scholars constantly encounter new challenges and generate new motivations, and then take some measures either actively or passively to achieve certain results. After reaching a balance, they generated new motivations and entered the next cycle. This leads to our second specific research question, which seeks to understand their motivations, what kind of strategies they adopted to deal with problems, and what results they achieved, that is, driver–strategy–outcome. Further, we combined motivation, strategy, results, and process stages. We marked the potential stages the interviewees mentioned, and finally deleted stages with few occurrences. In this sample, more than 80 percent of art scholars experienced all 7 stages. We named each stage according to the characteristics of the art scholars’ jobs, the content of their work, and their deep interaction with culture. Finally, after continuous discussion, analysis, and comparison, we named this process the cross-cultural reconstruction process, to describe the process by which art scholars reconstructed their own culture through in-depth cultural intervention in the cross-cultural context.

#### Open Coding

As required for open coding, we continued to collect data while analyzing them. We encoded already obtained qualitative materials and analyzed the art scholars’ home country study experiences and their life- and work-related experiences abroad. We coded the data line by line, naming each of the phenomena, events, actions, and meanings. The most important or frequently occurring codes were then condensed into concepts, which were further classified into categories. Based on the literature review, some categories were abstracted from the text data through labeling and repeated sorting and analysis, to create the initial coding basket. The original and new coding were compared, and this coding basket was continuously fine-tuned. Similar types of coding were combined to form advanced coding using continuous comparison (Glaser and Strauss, 1967; Charmaz, 2014). After the 26th interview, no additional categories were generated; three more materials were collected for confirmation. This analysis revealed distinct stages in the contact between this cross-cultural group and other cultures.

#### Axial Coding

This stage involved a clustering analysis of the independent data from open coding to establishing associations, selecting and building the main categories’ content, and connecting the main concept genera (Strauss and Corbin, 1998). The main category helped us understand the context for the development of events. Therefore, by analyzing the main axis, we identified different driving forces, strategies, and outcomes involving cross-cultural art scholars at each potential stage.

#### Selective Coding

Using axial coding, the relationship between the categories was further classified systematically, and the core categories (Strauss and Corbin, 1998) were condensed. This article constructs and develops a theoretical framework for the deep, cross-cultural remolding process of art scholars, through induction and refinement. It also describes the whole process from their initial attraction and aspiration to the shock and pain of entering a Western heterogeneous cultural environment, and then exploring, remodeling, and finally achieving integration; the two sub-processes of cultural adaptation and cultural integration comprise seven stages: cultural admiration, shock, adaptation, stranding, excavation, recombination, and integration (see Supplementary Table 2). The ladder model of the deep cross-cultural reconstruction process will be explained in the results section.

#### Research Quality Evaluation and Promotion

The two authors coded independently to test different coders; a team comprising all the students of the authors’ doctoral supervisor discussed the coding and the corresponding original text, thereby keeping the coding consistent and improving consistency between coding and text. After the first draft was completed, peer researchers were invited to review the coding results (Lincoln and Guba, 1985). We retained consistent codes and deleted inconsistent codes after discussion. Upon basic determination of the analysis framework, the verbatim versions of existing interviews were reviewed to further optimize the coding framework, ensure theoretical saturation of coding, and use the remaining three interviews to verify the theory (Locke, 2005).

## Results

By analyzing qualitative materials, we determined that the art scholars experienced two sub-processes during deep cross-cultural reconstruction: cultural adaptation and (more importantly) cultural integration. Cultural adaptation mainly refers to the process of social-cultural intervention and preliminary cognition of intellectual culture, including the three stages of cultural admiration, shock, and adaptation. Cultural integration refers to the process of deep cultural intervention, including the four stages of cultural stranding, excavation, recombination, and integration. This reconstruction gradually presents a step-by-step development process in accordance with cultural involvement, allowing art scholars to gradually reach a higher intellectual and cultural level. After experiencing these processes, the cross-cultural participants completed cultural reconstruction and gradually achieved cultural self-identification. In the following sections, Supplementary Tables 3–9 present typical descriptions of the interview contents relevant to each stage.

**FIGURE 1:**
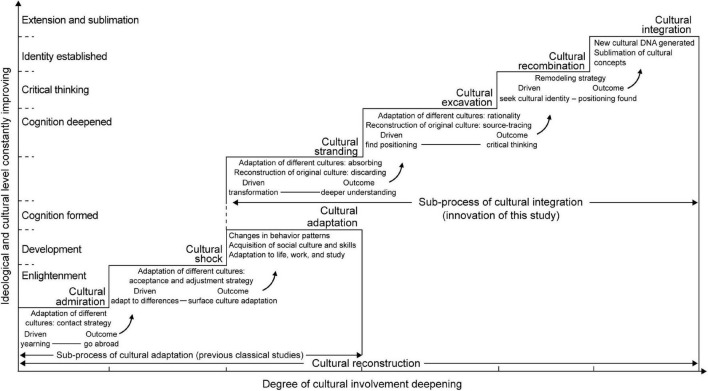
Ladder model of deep cross-cultural reconstruction process among art scholars.

Simultaneously, this study identifies that each process stage can be launched in line with the logic of driver–strategy–outcome, as triggered by motivation or conflict. This necessitates the application of a two-leveled strategy comprising the adaptation to a different culture and the remolding of the home culture to leap up the stages. The ladder theory of the deep cross-cultural reconstruction process proposed in this study breaks through the adaptation mode discussed in previous studies, based on the strong culture’s parallel or downward absorption (Figure 1). Therefore, this paper innovatively suggests the integration of two cultures separated by a great cultural distance in deep cross-cultural reconstruction, which is significant for the intellectual and, therefore, career growth and development of Chinese art scholars.

### Sub-Process of Cultural Adaptation

This sub-process is where the art scholars experience changes in their social life adaptability amid a unilateral acceptance of a different culture, and gain preliminary cognition of the different country’s intellectual culture. It comes in three stages: cultural admiration, shock, and adaptation.

#### Cultural Admiration Stage: Yearning and Initial Contact

Cultural admiration here entails (Chinese artists’ and art scholars’) initial contact with Western art and culture and the intense curiosity and yearning for cross-cultural learning. Generally, the motivation for this stage is triggered when art scholars encounter Western masters’ works of art and are deeply attracted to and admire their avant-garde artistic views and styles, thus self-generating strong curiosity and desire to learn (see Supplementary Table 3).

Typical description:

*I started painting expressionism before I graduated from college. At that time, there was no such atmosphere in China, and there were not many people who painted such exaggerated paintings in the academic system. I always hoped to have the opportunity to study in the hometown of expressionism, such as Germany. I liked Beckmann’s way of telling the story. (LD)*

The main strategy at this stage is a contact strategy. At this time, the artists had not connected with Western culture closely and systematically, but because they longed for it, they collect as much information about Western art as possible, even if fragmented. However, this stage did not involve reconstructing the home culture. In terms of adapting to a different culture, they used the available resources of people and cultural matters to implement their contact strategies. In terms of people, they sought more information on interpretation of host cultures and on-going abroad by consulting with teachers and senior schoolmates with experience of traveling abroad; in terms of cultural matters, they indirectly felt the beauty of Western art by attending school exhibitions and borrowing Western books, which were scarcely available in China until the 1990s.

Typical description:

*I always wanted to do something else, learn Van Gogh, Cezanne. I even drew light effects. At that time, I wanted to learn everything I could read in magazines. (ML)*

In the cultural admiration stage, art scholars were eager for a free artistic environment abroad and to embrace the avant-garde artistic concepts of the West. Eagerly they adopted a contact strategy to indirectly grasp Western art under constrained conditions while actively seeking opportunities to study abroad, to successfully realize cross-cultural learning. This stage in the cross-cultural process has rarely been mentioned, but is the driving force and premise of deep cross-cultural reconstruction.

#### Cultural Shock Stage: Feeling Shock and Contact Adjustment

Cultural shock refers to the process by which different thoughts on culture and art intrude on the cultural system, generating great shock that provides initial influence. This stage is similar to the honeymoon and crisis stages in the previous U-curve and W-curve models (Oberg, 1960; Gullahorn and Gullahorn, 1963; see Supplementary Table 4).

Typical descriptions:

*It was the first time I came into contact with contemporary art so directly. I didn’t know the cause and effect of anything, such as installation and video art; I couldn’t forget the strong visual impact of those works. (CRB)*

*When the professor gave me an offer as his student, he said “your works are very good, but it is totally outdated in our place; it is from many years ago.” (RR)*

This stage was mainly motivated by the desire of cross-cultural experiencers (now physically in the foreign cultural environment) to promote deeper understanding of a different culture. At that time, the information to which participants were exposed was no longer fragmented, and the resources of a different culture filled their entire learning, working, and living spaces, inspiring their strong desire for further learning and understanding. Simultaneously, the shock from exposure to information on the a different culture made them aware of their own lack of knowledge. As compared to the time when they set off for foreign countries, the scholars had a deeper sense of the huge differences in the material level and cultural influence caused by the economic development gap between developing and developed countries. They needed to first adapt to the new living, learning, and working environment, and establish social contact.

In this stage, they adopted the acceptance and adjustment strategy. Because the art scholars were still absorbing and digesting considerable information on the host culture, they were not reconstructing their home culture. Because of their short time in it, and eagerness to adapt to life in the new culture, the scholars seemed to willing accept the new information to which they were exposed. Further, to better adapt to the new environment, they socialized and interacted with their classmates for interpersonal experience; regarding cultural matters, they also learned languages, walked around to become familiar with the surroundings, broadened their horizons, and worked part-time outside school to experience life in that culture.

In the cultural shock stage, the scholars were initially involved in different cultural situations, and the strong emergence of cultural differences revealed a new world to them. The complex sense of discovery and short-term excitement made them perceive a backwardness of their home country’s economy and material poverty, their own ignorance of contemporary art, and consequently, cultural inferiority—feeling inferior to others in terms of cultural knowledge and level; however, this inferiority gradually faded with more time spent studying abroad, as shown in the sample. Faced by cultural shock, they adopted the strategy of passive acceptance and active adjustment. Some individuals were inspired by patriotism, stemming from feeling the gap between national pride—when looking back on their home country’s glorious history—and the decline of China’s current soft cultural influence. At this time, the profound cultural differences were the tip of the iceberg, but understanding and adapting to this gap were not yet achievable. This stage of understanding ended with social-cultural adaptation. After the feeling of freshness had passed, they faced a different experience and entered the next stage of cultural reconstruction.

#### Cultural Adaptation Stage: Cultural Acquisition and Life Adaptation

Cultural adaptation refers to the process of immersive contact with a different culture; continuous adjustment to cultural conflicts and role expectations; changes in and adaptation of behavioral patterns and adaptation; and the acquisition of social culture and skills to adapt to life, learning, and work (see Supplementary Table 5).

In previous studies, this marked the end of the process of adapting to host cultures. As previous models did not involve the internalization, intervention, and output of intellectual culture, it seems that—as a solution to material difficulties, poor communication, customs differences, or disharmony of interpersonal communication—the adaptation of learning (for students in disciplines such as science, industry, agriculture, commerce, and medicine), work, and life can be achieved. The research participants’ adaptation does not stop here, although some interviewees mentioned fellow cross-cultural learners stopping after reaching cultural adaptation. These learners adopted deep cultural learning (i.e., they came to learn philosophy, literature, and art) and involvement for going abroad; nevertheless, with the difficulties and temptations of real life, they lost the higher cultural pursuit. Some became street artists or entered art institutions as technical art teachers, turning to practical arts; some married local Germans and chose to switch careers. Their level of cultural adaptation did not require in-depth thinking about the underlying logic of the culture. These immigrants eventually learned the experience and ways of host cultures in survival practices, integrated into local cultural forms, and realized cultural adaptation in the universal sense described in the literature—achieving a better life and realizing their life values. Conclusion of the cultural adaptation stage can facilitate immigrants and many overseas scholars gaining closure as their ability to adapt is sufficient to cope with problems encountered while studying and working. However, this is a stepping-stone for the art scholars studied in this paper. They initiated a challenging cultural journey toward the significance of art history—a journey not mentioned in previous studies but becoming more significant.

### Sub-Process of Cultural Integration

This sub-process is where the art scholars eliminate the barriers between their home culture and the host culture, nurture new cultural concepts (based on integrating, reorganizing, and extending the two cultures), and influence the creation of new cultural works. This process encompasses four stages: cultural stranding, excavation, recombination, and integration.

It should be noted that deep, cross-cultural adaptation is not a universal phenomenon. Under the continuous deepening process, not all artists can enter the new process of cultural integration, which requires deep cultural intervention and reaching a deeper cross-cultural understanding. This sub-process differs fundamentally from the previous cultural adaptation sub-process because it is not influenced by the material level and rises to the mental level. Those scholars with higher cultural and artistic pursuits perceived great potential energy in Chinese culture. Their strong feelings toward family and country and their pursuit of Chinese cultural renaissance prompted them to pursue new journeys, experiencing cultural transformation in self-awakening. They are elites in the process of cultural reconstruction. Ultimately, most people will choose to return to China as a cultural builder. Even if some stay abroad, they also play the role of “migratory birds” and messengers, devoting themselves to revitalizing Chinese culture.

#### Cultural Stranding Stage: Cultural Collision, Absorption, and Discarding

The first stage in the sub-process of cultural integration is cultural stranding, which coincides with the cultural adaptation of the previous stage. These two stages are the transition from shallow to deep cultural intervention. Upon achieving adaptation in life and work, art scholars realized there was a clear and insurmountable boundary between the two cultures, such that in literary and artistic creation they faced difficulties integrating themselves into the mental and intellectual fields (see Supplementary Table 6).

Cultural stranding means that, as the understanding of a different culture gradually deepens, profound differences between the home and host cultures begin to emerge and internally collide within the art scholars. The two were difficult to integrate, causing pain, confusion, and stagnation in creative works. This stage differs from the previous U-curve and W-curve.

The motivation at this stage stemmed from our research participants’ need for deep cultural reconstruction. The cognitive gap in the consumer culture based on material income was easy to overcome. After adapting to life-work-based social culture, they grappled with the formidable obstacle of reconstruction in the mental and intellectual fields arising from the intellectual culture of the host country. Given the vast differences between Eastern and Western aesthetics, philosophical systems, and ways of thinking, the artistic creation styles formed by the artists and widely recognized in China were difficult for Westerners to appreciate, understand, and accept, according to the participants. Similarly, the scholars could not quickly grasp the essence of Western art. Under the dual gaps in the senses of identity and accomplishment, they faced difficulties in transformation and felt cultural loneliness from incompatibility; they lost motivation to create and fell into confusion and pain. No previous cultural adaptation model has covered this stage.

Typical descriptions:

*In China I was so admired, and in Germany I showed off my skills, but my professor didn’t even look at it. One time he did a calligraphy work, and he used his long shoelaces to paint on the shoes, and showed it to me, said look, this is my calligraphy. I said, only the shape resembles Chinese calligraphy, but it does not have the essence of Chinese calligraphy. He smiled and said so does your painting. It hit me very hard. (XJ)*

*Because you’ve been away from China for a few years, and it’s like your energy has been diluted away, and you’re in a vacuum. You’re not as nourished as you used to be, and you’re in a vacuum in a foreign culture. (SZ)*

At this time, the strategy adopted by the artists entailed absorbing and discarding influences at different levels. Pressured by cultural reconstruction’s demands (the “push” of host cultures) and resistance (the “pull” of the home culture), their strategy involved adapting to host cultures and reconstructing the home culture. The first strategic element entailed learning: in the interpersonal sense, they conducted culture and art discussions with professors, mentors, local classmates, and friends; in the cultural sense, they viewed countless exhibitions in European museums and galleries and widened their perspective by imitating masters’ works and adding Western elements (in terms of use of materials, way of narrative, etc.). The second strategic element entailed abandoning the satisfaction and vanity regarding their achievements in China and the technically exquisite concepts on creation and artistic styles they had pursued and established. In attempting to change their conceptions, they tried their best to re-adjust to their initial state, thereby truly reaching the creative level of contemporary art.

Typical description:

*That is the 2007 Venice Biennale, Kassel Documenta, and Mons sculpture exhibition: these three exhibitions were held about the same time. I just ran to a lot of places to see exhibitions. Also, I went to the Orsay Museum, [and saw] art of the Pompidou Center. In fact, I didn’t understand at first—what I knew was to see as much as I could. What impressed me most at that time was Anselm Kiefer’s retrospective exhibition in the Grand Palais in Paris, which affected me a lot. (LH)*

Because previously studied groups did not have to engage in deep cultural reconstruction, the cultural stranding stage was not proposed in previous models. However, this stage was crucial for the cultural reconstruction of artists who required deep cultural intervention. In the cultural stranding stage, they deeply felt the faults in Chinese contemporary art education and artistic concepts (the system is antiquated, lacks many fields as majors, and only emphasizes painting technology, with not enough emphasis on freedom and creativity), which made it difficult to achieve mutual understanding with other cultures. They were immensely troubled by integration difficulties and cultural loneliness. They felt unappreciated and unsupported, and thought their cultural ideas were not understood. However, in this period of confusion and pain, the scholars drew on the absorbing and discarding strategies and gradually gained deeper knowledge and understanding of the a different culture in which they were located. They tried to “unlearn to start over” and looked for a gateway to Western-style creativity by imitating the Western masters’ works.

#### Cultural Excavation Stage: Lack of Orientation and Rational Source-Tracing

Cultural excavation is the process through which the scholars began to seek a post-confusion breakthrough by absorbing the host culture and reconstructing their home culture. They sought to balance contradictions, and to find a new director for their artistic creativity. This stage was not included in previous cross-cultural adaptation models (see Supplementary Table 7).

Artists at this stage had a strong motivation for new exploration. In practice, they realized that the one-way input of a different culture caused a lack of personal reflection and self-positioning in their work. Imitating the style of Chinese and Western masters, adding elements of Western culture, and playing to the trend toward Western standards did not arouse the appreciation and interest of the Western art market. Additionally, the style they had created might not have embodied the true essence of Eastern culture in the way they had previously thought. Consequently, they began to reflect more consciously on rationally tracing the origins of the host and home cultures, reconstructing their cognition of the two cultures, and exploring their own artistic style on this basis.

Typical description:

*The professor said your lines were Michelangelo’s. We learned from others, and we have never thought about whose lines they were. That’s how we used to do it. But there is a purity in German art, and they can see your character in the lines. (WCY)*

At this stage, their strategy entailed reasoning and source-tracing. They found, through feedback from professors, mentors, classmates, and audience of exhibitions in and out of school, that their work lacked deep cultural connotations and a salient personal style—this was almost a universal experience in our sample. Urgently needing to find their own position, they adopted a strategy of reasoning for adapting to host cultures. Interpersonally, they reflected rationally and critically on that feedback from others. They were guided by professors and mentors to objectively view their predecessors’ works, avoid the convergence of artistic creativity, and actively contact artists from various countries to expand their artistic horizons and absorb different cultural insights. Culturally, they consciously studied and absorbed the culture, gained insight into the essence of Western artistic thought, critically studied the masters’ works, grasped the essence of Western artistic culture, and abandoned the simple imitation of Western elements. In reconstructing the home culture, they re-examined the Chinese culture, which they did not entirely understand, and adopted a source-tracing strategy. Interpersonally, they actively sought to communicate with individuals possessing profound insights into Chinese culture, including many foreign professors and mentors. Culturally, they began to dabble in traditional Chinese books, studied Chinese philosophy, and sought new ideas and breakthroughs.

Typical descriptions:

*I studied a lot of Western art theories at that time, including Newman and Reinhart, and then, I became interested in many thoughts [on art] in ancient China, such as those in Wei and Jin Dynasties. (CRB)*

*What’s true and what’s right? Some professors hold this view and other people see it in that way, it doesn’t matter. We need to become an independent person, not dependent on the opinions of others. This independence produces a kind of artistic self-judgment. (SZ)*

At the cultural excavation stage, with reasoning and source-tracing strategies through a two-way exploration of other cultures, the scholars formed a deep understanding of and critical insights into a different culture. The remodeling of the relationship was ready.

#### Cultural Recombination Stage: Blending *via* Remodeling and Finding the Position

Cultural recombination refers to the process in which—after deep-diving to explore and excavate the home and host cultures and implement re-understanding by balancing and selecting—the scholars developed cognitive complexity and structurally improved their capabilities of processing home and foreign cultural information, on which basis they reorganized Chinese and Western cultures, constructed new cultural DNA, and formed new cultural patterns and individualized artistic languages. Previous models have not addressed this stage at all (see Supplementary Table 8).

Typical description:

*The reason why art has its value is to drill into the depth of culture and the heart of people. The two different cultures share the same research. The European was able to repeat their own styles because they spent their whole life building their symbols, to combine something new into it, and it was what they saw all their lives, not to take something from someone else, which is very different from what we did before. Everyone is different, you just have to keep your eyes on the world, connect your whole culture, and that’s a kind of DNA. (WCY)*

Motivation at this stage was due to the artists’ new understanding of the home and host cultures after experiencing the collision with and exploration of other cultures; under this new understanding, they found that the two were no longer contradictory. However, there remained some ambiguity in their self-identification in culture, and it was necessary to choose and balance between the two cultures for a unified and organic practice. This entailed resolving the conflicts between the different cultures’ innate expressions, reorganizing the innate expressions from the home and host cultures in their personal art systems, and forming a personalized artistic style and cultural perspective.

Therefore, the strategy at this stage involved remodeling: the individuals attempted a series of actions to restore internal balance. To this end, the scholars selected, extracted, and redistributed the two cultures in the system and regrouped them into new cultural DNA. They realized that the two cultures were not mutually exclusive, and that self-identification with a culture no longer required them to have any particular traits. Consequently, they entered the final stage of cross-cultural reconstruction: cultural integration.

In the cultural recombination stage, the art scholars remodeled and changed their materials, ways of expression, and content through strategies, forming new cultural DNA, thereby moving up to cultural integration. This breakthrough laid the foundation for their subsequent development.

#### Cultural Integration Stage: Sublimation of New Cultural Concepts

Cultural integration is the process whereby art scholars deeply extracted and merged Chinese and Western cultures that have affected each other, generated new cultures by combining the two cultural perspectives, created cultural content, and exerted cultural influence. This pushes our cross-cultural reconstruction model to the highest stage, which is also where social-cultural adaptation models cannot exist. However, not all artists can achieve such a leap (see Supplementary Table 9).

Those artists who were able to realize cultural remodeling strategies balanced, merged, and sublimated the Chinese and Western cultures in their framework, recognizing the differences between them. They transformed their deep understanding of Chinese and Western cultures into inspiration and motivation for artistic creation. Culture and art were examined at a new level. During this stage, the cross-cultural experience of learning Western art fully demonstrated its value and influence on the scholars, sublimating them in the understanding and cognition of culture. The cross-cultural reconstruction process of the focal group ended with cultural integration, and scholars’ cultural perception entered another higher level—with individuals’ immensely improved perception abilities, thinking, and insight related to seizing the essence and discovering the commonality of home and host cultures.

Typical description:

*At that time, I found that the East and the West actually had something in common in the mental realm, that is, in the realm of free spirit. (CRB)*

In summary, the deep cross-cultural reconstruction of art scholars comprises two linked sub-processes of cultural adaptation and cultural integration. Furthermore, cultural admiration, shock, and adaptation constitute the sub-process of cultural adaptation confined to social-cultural intervention, whereas cultural stranding, excavation, recombination, and integration constitute the sub-process of cultural integration involving deep cultural intervention.

## Discussion

In cross-cultural research, despite decades of fruitful research results across different disciplines, there remains a problem of knowledge disconnection. Scholars have conducted several studies from the perspectives of psychology, communication, and sociology, whose different foci make it difficult to form a clear and coherent interdisciplinary knowledge system for cross-cultural research. In anthropological and sociological studies, concepts such as the stranger—the free wanderer (e.g., Simmel, 1950)—the marginal man—a cultural hybrid without a sense of identity (Park, 1928)—and the sojourner—a cultural other without assimilation (Siu, 1952)—were developed, and the process of cross-cultural adaptation proposed (Lysgaard, 1955; Oberg, 1960; Gullahorn and Gullahorn, 1963; Kim, 2017). In social psychology, scholars estimated the degree and results of cross-cultural adaptation according to recognition of cultural identity and adaptation attitude, and then developed theories of levels (Searle and Ward, 1990; Kim, 2017) and strategies (Berry, 1976; Bourhis et al., 1997) of cross-cultural adaptation. However, the cross-cultural adaptation process of cultural and artistic talents has not been considered. Moreover, the process stages, levels, and strategies of cross-cultural adaptation are independently researched, with no integrated study of these closely related parts. This study attempts to fill this theoretical gap, taking art scholars as the research participants, analyzing the deep cross-cultural reconstruction issues, and combining the adaptive levels and strategies in the process to form an integrated model.

Previous literature has been classified as taking the perspective of “assimilation,” “acculturation,” “alternation,” “multicultural,” or “fusion” when discussing biculturalism. According to LaFromboise et al. (1993), “assimilation” and “acculturation” focus on the acquisition of the host culture, emphasizing a linear and unidirectional relationship between the home culture and host culture. Under “assimilation,” through acquisition they will form the identity of host culture, but under “acculturation” will always be identified as a member of their home culture. “Alternation” posits a bidirectional and orthogonal relationship between the individual’s home culture and host culture. It urges us to consider the influence that individuals from both cultures have on each other. The “multicultural” view provides a pluralistic approach to considering the relationship between two or more cultures that are tied together within a single social structure. It addresses the possibility of cultures maintaining distinguishing identities, such that individuals could be involved in one culture while maintaining their identity in another. “Fusion” emphasizes the production of a new culture and assigns equal status to the two or more cultures. However, the “reconstruction” view discussed in this research shows the development of artists and art scholars from a linear and unidirectional process of acquisition to a bidirectional and orthogonal balance, and thence to reconstructing the cultural formation and producing a cultural identity. Living abroad benefits individuals by facilitating enhanced creativity and integrative complexity, but the issue of how one takes a bicultural approach while abroad can be critical to producing lasting cognitive changes and psychological benefits (Tadmor et al., 2012).

The art scholars’ deep cross-cultural reconstruction is a process of deepening and sublimating culture through in-depth development of personal cultural connotations, with a longer cycle, more complicated stages, and a more profound degree of intervention. Generally, previous process studies have summarized models according to individuals’ psychological feelings toward and interactions with other cultures. Nevertheless, the important category of art scholars has been overlooked; previous cross-cultural adaptation process models cannot fully explain the series of conflicts, contradictions, and struggles this group experiences in the cross-cultural process.

This study focuses on the process of cross-cultural adaptation (or, more precisely, the process of cross-cultural reconstruction). Therefore, we reviewed three classic studies of cross-cultural process research: the U-curve theory (Oberg, 1954), W-curve hypothesis (Gullahorn and Gullahorn, 1963), and stress–adaptation–growth dynamic (Kim, 2017). Although these studies are controversial, many subsequent studies on the cross-cultural process have been conducted on this basis. However, different forms have been studied at the social-cultural level, including with regard to the scope of adaptation to the physical environment, conditions of social–material life, economic conditions, social systems, interpersonal communication, and cultural norms and practices related to life and work. The artists’ reconstruction model is not only about individuals’ psychological feelings and general social-cultural adaptation but also includes the depth of cultural participation and involvement and eventually achieving cultural remodeling and integration. Thus, the focus of this article is the difference between the artist’s reconstruction model and these classic models.

In terms of the ladder model, the logic of “driver–strategy–outcome” exists in each stage of the reconstruction process; as the degree of cultural involvement continues to deepen, different levels of cultural factors will trigger different motivations—further impacting the strategies and results. Moreover, for art scholars, their strategy is not unilateral cultural absorption, but rather consideration of the two levels of adaptation to a different culture and reconstruction of the home culture based on the new perspective gained abroad.

The transition from a less influential to a stronger culture must differ in terms of the stages and difficulties of the transition, as compared to the previous parallel or downward absorption adaptation model; the ladder model can better present the entire process. For instance, along with the continuous deep intervention of culture, the bottom-up, deep, cross-cultural reconstruction model shows the transition from worship to disenchantment and, finally, complete self-establishment, incorporating full mental and intellectual sublimation.

### Significance

This study’s results offer a detailed explanation of the deep, cross-cultural reconstruction process of art scholars under different cultural potentials, providing detailed and accurate descriptions of relevant processes and mechanisms. The study also discovers these cross-cultural artists’ strong desire for cultural reconstruction; it describes their acts of comparison, research, and analysis of the Western contemporary and Chinese art systems.

This study can also be placed in the framework of biculturalism, with art scholars appearing as a distinct group that deeply involves itself with an internalizes culture and works on cultural reconstruction and cultural formation; thus, our work describes a distinct phenomenon within cross-cultural research.

Besides enriching theoretical understanding, this study also offers practical guidance. First, at the individual level, the ladder theory guides deep cross-cultural groups to form a culturally dual response psychologically, physically, and emotionally. Psychologically, the shocks they face are far stronger than those faced by ordinary survivors of cultural shock, because the complexity of the cultural engagement they face and hence their confusion is constantly deepening. Therefore, it should account sufficiently for psychological construction. Moreover, when facing a different culture, one should neither practice blind servility to foreign things nor blindly exclude them—both responses that are not unfamiliar in cross-cultural engagement; instead, one must use foreign things while engaging in dialectical choices. Physically, one must actively seek available resources, including literature, philosophy, aesthetics, and people with deeper understanding and cognition of intellectual culture, while also expanding deep learning and communication. Emotionally, one must be prepared to face loneliness and self-identity issues as an outsider. Host cultures should not be regarded as the opposite of home cultures, however; people should also try to understand the effect of their own cultural backgrounds and national memories on existing cultural differences and their impact on current cultural exchanges. Concurrently, cross-cultural individuals must develop the ability of cultural empathy and understand the perspective of a different culture.

Second, at the social level, the cross-cultural support system founded on schools and related social institutions should be further improved. In the education system, schools should strengthen intellectual education on multicultural knowledge, especially philosophy and aesthetic education, and guide students to practice critical, dialectical thinking. Furthermore, social institutions such as museums, art galleries, and libraries should actively introduce high-level literature and artworks from around the world, hold exhibitions and cross-cultural forums, provide learning resources, and build communication platforms. A two-pronged approach to the individual response and social support system will help resolve the dilemma that strategies cannot be effectively implemented in the cross-cultural process, and establish cultural consciousness and self-confidence.

## Limitations and Future Directions

Although our conclusions improve explanatory and practical guidance for cross-cultural process theory, the validity of the generated theory should be tested through subsequent large sample data. Additionally, our research sample creates certain limitations as only one country and culture of study were considered; the convergence of sample occupations is also a limitation. The homogeneity of samples is a key characteristic of qualitative research. It is only in a specific sample that reliable, regular conclusions can be drawn, so researchers limit the scope of particular work accordingly. The current reconstruction model reflects the conclusions drawn by famous contemporary artists in China. However, this homogeneity is also a limitation, and considering other types of scholars is the next step of our research. We will select more diverse research samples to verify whether other types of people and their experiences are in line with this reconstruction model. Besides, future studies could expand the scope of destination countries and occupation types. Other research avenues include studying: the impact of individual traits and cultural orientation on art scholars’ deep cross-cultural reconstruction; the relationship between cultural reconstruction and these scholars’ career development and mechanism of influence; and differences in the cognition of host and home cultures between scholars who have experienced the cross-cultural process and those who have not.

## Data Availability Statement

The original contributions presented in the study are included in the article/Supplementary Material, further inquiries can be directed to the corresponding author/s.

## Ethics Statement

Ethical review and approval was not required for the study on human participants in accordance with the local legislation and institutional requirements. All participants gave written informed consent in accordance with the Declaration of Helsinki. Written informed consent was obtained from the individual(s) for the publication of any potentially identifiable data included in this article.

## Author Contributions

FZ analyzed the data and wrote the manuscript under the guidance of JX. JX formulated this study and contributed to editing of the manuscript and critical revisions. JX, LL, and FZ conducted the interviews. LL contributed to study design and data analysis. All authors contributed to the article and approved the submitted version.

## Conflict of Interest

The authors declare that the research was conducted in the absence of any commercial or financial relationships that could be construed as a potential conflict of interest.

## Publisher’s Note

All claims expressed in this article are solely those of the authors and do not necessarily represent those of their affiliated organizations, or those of the publisher, the editors and the reviewers. Any product that may be evaluated in this article, or claim that may be made by its manufacturer, is not guaranteed or endorsed by the publisher.
