# Supplementary material for: Deep Cross-Cultural Reconstruction Process in the Context of Cultural Potential: A Qualitative Study
Source: Front Psychol. 2022 May 6;13:727616. doi: 10.3389/fpsyg.2022.727616 (PMC9120592; doi:10.3389/fpsyg.2022.727616)
Supplement: Supplementary file 1 [file Data_Sheet_1.pdf]

Supplementary Materials

Supplementary Table S1. Basic information of interviewees.

No.	Code	Year of birth	Before studying abroad	Year going abroad	Year of return	Residence	Occupation	Interview (mins.)	Words
1	CL	1982	Undergraduate	2007	2011	China	Freelance artist	181	32,367
2	CRB	1970	Undergraduate	1992	1998	Germany & China	Freelance artist	74	10,424
3	CYF	1962	Museum staff	1997	1999	China	Associate professor	96	15,449
4	DGY	1957	Teacher	1995	1998	China	Dean, professor	106	18,674
5	LD	1963	Teacher	1990	1995	China	Freelance artist	136	25,412
6	LH	1984	Freelance artist	2009	2010	China	Freelance artist	87	14,773
7	LJ	1963	Freelance artist	1989	2006	China	Freelance artist	152	27,391
8	LYG	1964	Teacher	1992	1997	Germany & China	Freelance artist	161	28,195
9	ML	1958	Undergraduate	1982	1984	China	Associate director, professor	102	18,754
10	MSQ	1956	Teacher	1989	1995	France & China	Freelance artist	98	17,386
11	MXC	1964	Freelance artist	1995	1999	China	Professor	95	16,739
12	QAX	1972	Freelance artist	1998	2003	China	Freelance artist	83	14,744

13	RR	1960	Undergraduate	1986	n/a	Germany & China	Professor	104	17,285
14	SZ	1965	Government staff	1991	2003	China	Associate dean, professor	124	19,634
15	TP	1960	Teacher	1989	1994	China	Associate dean, Professor	108	18,111
16	TF	1963	Teacher	1990	1995	China	Professor	113	18,560
17	TX	1976	Undergraduate	2000	2005	China	Professor	121	20,300
18	WCY	1959	Teacher	1991	1995	Germany & China	Professor	153	25,409
19	WXH	1957	Teacher	1987	1992	Germany & China	Professor	87	15,715
20	WXS	1964	Designer	1990	2003	China	Professor	186	33,024
21	XH	1972	Designer	2004	2010	China	Associate professor	148	25,814
22	XJ	1955	Teacher	1988	1989	China	Dean, professor	102	17,089
23	YJS	1955	Freelance artist	1991	1996	China	Curator, professor	98	17,424
24	YQ	1952	Teacher	1987	n/a	Germany & China	Professor	162	28,561
25	YCG	1955	Teacher	1990	1997	China	Director, professor	179	29,037
26	YXZ	1976	Trainer	2002	2006	China	Professor	147	25,129
27	ZGL	1957	Teacher	1991	1997	China	Professor	95	16,941
28	ZJS	1954	Freelance artist	1986	1990	China	Freelance artist	87	15,868
29	ZQS	1957	Teacher	1990	1995	China	Director, professor	146	25,157

Supplementary Table S2. Stage model of deep cross-cultural reconstruction among culture and art scholars.

Stage name	Stage connotation
Cultural admiration	The stage from the time of the art scholars' initial contact with Western art and culture to their intense curiosity and yearning for cross-cultural learning.
Cultural shock	The stage in which the thoughts of different cultures and arts invade the cultural system of the scholars, bringing about great shock and initial influence.
Cultural adaptation	The stage of immersive contact with a different culture, continuous adjustment to cultural conflicts and role expectations, changes in and adaptation of behavioral patterns, and the acquisition of social culture and skills for life–learning–work adaptation.
Cultural stranding	The stage in which, with gradually deepening understanding of different cultures, profound differences between the home and foreign cultures begin to appear, making integration difficult and causing stagnation and confusion.
Cultural excavation	The stage in which culture and art scholars begin to seek a breakthrough after confusion; they continue to explore in two directions—absorption of different cultures and reconstruction of the original culture—seeking balance in contradictions and a new direction in their art creation.
Cultural recombination	The stage in which, after developing cognitive complexity and structurally improving their abilities to process home and foreign cultural information, cultural and art scholars reorganize Chinese and Western cultures, construct new cultural DNA, and form new cultural patterns and individualized artistic languages.
Cultural integration	The stage by which culture and art scholars deeply extract, integrate, and generate new culture from the Chinese and Western cultures that affect them, creating cultural content and exerting cultural influence from the perspectives of both cultures.

Supplementary Table S3. Driver–strategy–outcome in cultural admiration stage.

Cultural admiration	Conceptualization		Typical descriptions in the original text
Driver	Attracted by different cultures		"I always thought it must be very mysterious, so I want to go abroad." (ML)
			"I want to learn more and optimize myself." (SZ)
			"I desire to understand Western culture deeper." (CYF)
Strategy	Adaptation of different cultures: Contact strategy	Interpersonal: Consult teachers and seniors with overseas experience	"I was interested in those teachers who came back from Germany, and then became friends with them." (CL)
			"At that time, after returning from Germany, Ma Lu made an exhibition, which touched me a lot." (LD)
		Cultural matters: Attend exhibitions on campus and borrow books and album of painting	"I got some information about Germany from books." (YXZ)
			"At that time, our school had many international exchange activities, exhibitions and lectures, and we often listened to them." (SZ)
	Reconstruction of the original culture	Temporarily not covered	
Outcome	Study abroad		"I took the train from Beijing, through Ulaanbaatar to Moscow, Moscow to Poland, Poland to East Germany, East Germany to West Germany." (WXS)
			"I went to Germany just after I got married." (RR)

Supplementary Table S4. Driver–strategy–outcome in cultural shock stage.

Cultural shock	Conceptualization			Typical descriptions in the original text
Driver	Adapt to the differences			"When I got there, I went to the museum and realized it was different than I thought." (LYG)<br>"There was too much information when I got there, a lot of struggle, I felt confused." (XH)
Strategy	Adaptation of different cultures	Acceptance strategy	"Blindly" following the flood of information	"I didn't understand anything, but they impressed me and I was moved." (LH)<br>"I got feelings about new stuff too, then I pretended I was entering the flow state." (TP)
		Adjustment strategy	Interpersonal: Socialize with classmates	"At the beginning, I hardly dared open my mouth to talk, but I gradually communicated with my classmates." (ML)<br>"Before I could find any place suitable, I stayed with him [a German student named Leica]." (MSQ)
			Cultural matters: Learn language, take a part-time job, become familiar with the environment, and broaden horizons	"When you go to a strange country, language is the most important thing." (RR)<br>"At first I didn't know any better, so I went to many places by myself." (LH)<br>"Working to earn money is one thing, but to experience life is more important." (SZ)
	Reconstruction of the original culture		Temporarily not covered	
Outcome	Surface-structure sociocultural adaptation			"It opened up your mind and your freedom." (XH)<br>"My basic problem was language. In fact, once this was crossed, I didn't think there would be too big a problem in communication" (LD)

Supplementary Table S5. Driver–strategy–outcome in cultural adaptation stage.

Cultural adaptation	Conceptualization	Typical descriptions in the original text
Adaptive state	Changes in behavior patterns	"From the overall state, at that time my learning and adaptation to the society have reached a stage…my behavior actually changed a lot." (SZ)
	The acquisition of social culture and skills	"Some artists went out on the street and made a lot of money a day, which was definitely a huge temptation, but once you start on this road, you will not do art anymore…. However, through this you can relate to that society." (MXC)
	Adaptation to life, work, and study	"We're going to learn Western stuff, but it's superficial to just take and learn the result. In fact, there is always another thought behind the form, but it is often ignored; people do not always think deeply about its relationship to philosophy or even to science." (QAX)

Supplementary Table S6. Driver–strategy–outcome of the cultural stranding stage.

Cultural stranding	Conceptualization		Typical descriptions in the original text
Driver	Facing the need for transformation		"Due to the original background of Chinese ink painting, the transformation is still a long-term thing." (CRB)<br>"No one will pay much attention to it, so there is a sense of cultural loneliness in it." (DGY)<br>"At that time, I could not turn around and was still in pain." (CYF)
Strategy	Adaptation of different cultures: absorbing strategy	Interpersonal: Discuss art and culture with professors and classmates	"The professor said I think you should take it a step further, and I recommend two artists that you can do some research about." (LH)<br>"You can learn something by communicating with your classmates, sharing thoughts and ideas." (CL)
		Cultural matters: view exhibitions often, imitate masters, add Western elements	"I know almost all those paintings so well that I can tell who painted it if I look at it from a distance." (ML)<br>"Later on, I spent a lot of time looking at paintings in museums every day. I think museums helped me lay the foundation for my future life." (LH)
	Reconstruction of the original culture: discarding strategy	Discard previous techniques and styles	"So I spent a decade throwing away what I learned and then looking for myself." (WCY)<br>"You have to give up something to get something, and getting rid of something is painful." (CRB)
Outcome	A deeper understanding of the different cultures in which they live		"I don't need to care too much about cultural differences or conflicts, which will naturally form an individual recognition in you." (XH)<br>"I think those rigorous things and rational thinking in Germany are a kind of integration for me." (WXH)

Supplementary Table S7. Driver–strategy–outcome in cultural excavation stage.

Cultural excavation	Conceptualization		Typical descriptions in the original text
Driver	Find their own positioning based on the lack of positioning		"The professor said your lines were Michelangelo's. We learned from others and we have never thought about whose lines they were." (WCY)
			"My previous pieces looked a little bit like the work of someone else. Take a little inspiration from this artist and copy that artist's brushwork." (CL)
Strategy	Adaptation of different cultures: the rational strategy	Interpersonal: receive guidance from professors and mentors, and contact artists from all over the world	"The professor said that I have shown you so many picture books not for you to study, but to know what has already existed, so that you can recognize what we did wrong today." (WCY)
			"I also came into contact with artists from various countries and saw their working style, life attitude, and cultivation in the process of art." (CYF)
		Cultural matters: learn and absorb pertinently, abandon pure imitation	"This independence produces a kind of self-judgment. Other masters look at a person in their way but I can do it in my way; this is an individual thing." (SZ)
	Reconstruction of the original culture: source-tracing strategy	Interpersonal: discuss with individuals with deep insights into Chinese culture	"Professor Jerison Newke and I went to Huang Binhong's hometown. Later, we bought a very thick album of the complete works of Huang Binhong from the second-hand book market of Panjiayuan that had been published by People's Fine Arts Publishing House. From then on, I gradually began to pay attention to Chinese painting." (CL)
			"Lehrer is affectionate about China; he thinks China is a great country. He always mentions that people will have a different

			understanding about their own culture when they study abroad." (QAX)
		Cultural matters: dabble in traditional Chinese books	"I have revisited many of the thoughts of ancient China." (CRB)<br><br>"There was a bookstore near the Hamburg train station where I read a lot of traditional Chinese books and engaged in Chinese calligraphy and Chinese culture, including *xiangqi* [Chinese chess]." (XJ)
Outcome	Development of an objective and critical view of the two cultures		"At that time, I found that the East and the West actually had something in common in the spiritual realm." (CRB)<br><br>"He's a professor. He has his own opinions. It's hard to say whether they're right or wrong." (LJ)

Supplementary Table S8. Driver–strategy–outcome in cultural recombination stage.

Cultural recombination	Conceptualization	Typical descriptions in the original text
Driver	Due to the ambiguity of identity, it is necessary to seek cultural identity again	"Those were not my own. I didn't have a cognitive ability and other things to go far, go wide, and go deep." (TF)
		"You have to explore a person's entire culture in an artistic way. Each person's growth path is different, and you have to use your method to connect your entire history and find its form, which is a kind of DNA." (WCY)
Strategy	Remodeling strategy	"I've taken what I know and developed them into my own thing, creating my own feelings, that was the most important thing to do." (CL)
		"A lot of things are to be integrated, but not to put them together." (ML)
Outcome	Positioning has been found	"By that time, I was really feeling it." (CL)
		"What I expressed was my relationship to the world." (YQ)
		"After this kind of cultural comparison, I found that, in fact, there are many ways to view the world. Although you may be a little bit small, the value is still there." (YJS)

Supplementary Table S9. Driver–strategy–outcome of the cultural integration stage.

Cultural integration	Conceptualization	Typical descriptions in the original text
Integrated state	New cultural DNA is generated	"You don't have to think about being different, you just have your eyes on the world, and you dig in your way, stringing together your entire history, and you will find the form, and that's a kind of DNA."(WCY)
		"The truth is intertwined at a certain point; it's something common to all human beings, but approached in different ways, so I don't feel the need to deliberately emphasize the East and the West." (MSQ)
	The sublimation of cultural concepts	"I didn't put myself much within the confines of so-called ethnic areas at that time, because I knew that people are the most important, and people are the same all over the world." (XH)
		"So, in a sense, in the spirit of artistic creation, the essence is that you have to be free." (SZ)
		"At that time, I found that the East and the West actually had something in common in the spiritual realm, that is, in the realm of free spirit." (CRB)
